# Caution about overdiagnosis of neck calcification

**DOI:** 10.1002/jgf2.391

**Published:** 2020-10-26

**Authors:** Junki Mizumoto

**Affiliations:** ^1^ Department of Medical Education Studies International Research Center for Medical Education Graduate School of Medicine The University of Tokyo Tokyo Japan

To the Editor,

I read the article by Akaishi et al^1^ with great interest and strongly agree that diagnosis of benign conditions with minimal invasion is important. Among all, the diagnostic flowchart as shown in figure 2 is quite useful for many primary care physicians.

However, I concern that the chart may lead to misdiagnosis because it attaches too much value to imaging findings. Calcium deposition in soft tissue is not always symptomatic. For example, CT of cervical spine which was routinely undergone in a trauma center detected atlantoaxial calcium deposition in 35% and 49% of patients with age over 60 and 80, respectively.^2^ Although the prevalence of asymptomatic retropharyngeal calcification remains unclear, 7.5 to 20% of asymptomatic adults have radiologically evident calcification in the rotator cuff.^3^ Considering that calcification in the longus colli occurs with the same mechanism as in the shoulder or hip,^4^ it is estimated that asymptomatic retropharyngeal calcification is not rare. Then, physicians following the chart may reach a wrong diagnosis when patients with asymptomatic calcification develop severe conditions such as meningitis or cervical spondylitis, both of which do not present specific findings in CT.

I propose that two points should be added to the original chart. Firstly, if patients with acute painful stiff neck and no red flags have some imaging features of calcification, physicians should start anti‐inflammatory treatment and then follow up the patients closely. That is, a definite diagnosis should be postponed until symptoms subside. Secondly, if physicians do not have barriers to additional tests such as lumber puncture (eg house call), they should not hesitate to perform such tests in order to rule out life‐threatening conditions. It is especially important when the conditions of patients are inconsistent with the illness script of calcification (eg young age, antecedent infection, nonremission within one to two weeks.)

## CONFLICT OF INTEREST

The authors have stated explicitly that there are no conflicts of interest in connection with this article.
